# Didactic dissonance—embracing the tension between classroom and clinical education

**DOI:** 10.3389/fmed.2023.1197373

**Published:** 2023-06-22

**Authors:** Aram S. Mardian, Lisa Villarroel, Lori Kemper, Heidi E. Quist, Eric R. Hanson

**Affiliations:** ^1^Chronic Pain Wellness Center, Phoenix VA Health Care System, Phoenix, AZ, United States; ^2^Department of Family, Community and Preventive Medicine, University of Arizona College of Medicine–Phoenix, Phoenix, AZ, United States; ^3^Arizona Department of Health Services, Public Health Services, Phoenix, AZ, United States; ^4^Midwestern University, Arizona College of Osteopathic Medicine, Glendale AZ, United States; ^5^Department of Psychiatry, University of Arizona College of Medicine–Phoenix, Phoenix, AZ, United States

**Keywords:** didactic dissonance, hidden curriculum, pain education, clinical education, transformative learning, adult learning theory

## Abstract

The United States is undergoing a transformation in the way pain is viewed and treated. This transformation affects pain education, as some degree of disconnect will be expected between what is taught in classroom settings and what learners observe in clinical settings. We term this disconnect “didactic dissonance” and propose a novel process to harness it as a learning tool to further pain education. Based on principles of transformative learning theory, we describe a structured, three-step process beginning with (1) priming learners to recognize didactic dissonance and identify specific examples from their education, followed by (2) encouraging learners to search the primary literature to resolve observed dissonance and reflect on the system factors that created and perpetuated the disconnect, and then (3) providing an opportunity for learner reflection and planning for how they will address similar situations in future practice and teaching environments. Fostering an environment conducive to learning—through modeling the intellectual virtues of curiosity, humility, and creativity—is a critical task for educators when implementing this process. Recognizing challenges faced by educators in both classroom and clinical settings, it may be a more feasible first step to integrate the concept of didactic dissonance into existing curricular elements. For programs able to implement the full three-step process, a discussion guide along with an example of a facilitated discussion have been provided. While proposed in the context of pain education, this transformational approach can be utilized across all topics in medical education to foster autonomous lifelong learning.

## Introduction

Learning in both the classroom and clinical environments have long been key components of medical education. The storied 1910 Flexner Report established many of the present-day standard for the sequence of 2 years of basic science education in the classroom followed by 2 years of clinical education at the bedside (1, 2). An unintended consequence of this structure has been to create a separation between classroom and clinical education, which can contribute to a disconnect or even contradiction between what is taught in the classroom and what is taught during the rotations that comprise clinical education. This disconnect has been lamented as a problem that can degrade a high-quality education (3–6) and is exacerbated by changing cultural paradigms. Efforts to address this disconnect have often focused on reducing the gap by creating a more integrated curriculum (2) and introducing initiatives to enhance the transfer of classroom learning to the clinical settings (7). While these efforts are important, they are insufficient, as the constantly evolving state of medical knowledge and practice will always create some degree of disconnect in curricular content between classroom and clinical education.

This type of disconnect could be expected to particularly occur in pain education, as pain management and pain education are undergoing a cultural (8). For example, while learners may be taught about the lack of evidence for long-term opioid therapy for chronic pain in the classroom setting, they may see providers routinely starting opioid therapy in the clinical setting. Alternatively, a classroom curriculum might teach a one-size-fits-all approach to opioid tapering, while in clinic a learner might observe a complex and nuanced approach to it.

The term *didactic dissonance* was coined in the *Arizona Pain and Addiction Curriculum* [co-chaired by ASM and LV— (9)] and describes the disconnect learners experience between what is taught in the formal classroom setting and what is taught and observed in clinical practice, and we propose that it is essential to embrace as an educational tool.

The importance of addressing didactic dissonance was endorsed by the multidisciplinary authors of the *Arizona Pain and Addiction Curriculum* (9), a modern, evidence-based, public health-oriented curriculum that aims for statewide cultural transformation to address the dual challenges of chronic pain and addiction. The curriculum workgroup anticipated that learners who were exposed to the new curriculum would inevitably experience subtle to blatant didactic dissonance during their clinical rotations, and thus agreed that the curriculum should recommend harnessing this dissonance to reinforce principles of the formal curriculum. Upon evaluation of the curriculum’s implementation, however, schools reported that addressing didactic dissonance was difficult to implement (10).

In this paper, we hope to meet the challenge of harnessing didactic dissonance. We provide an implementation process that applies transformative learning theory (11) to leverage aspects of this disconnect as a pedagogical tool. Rather than seeking to avoid or eradicate this didactic dissonance, we propose a method to embrace it while reinforcing key intellectual virtues to foster lifelong learning and information mastery.

## Defining didactic dissonance

The term didactic dissonance combines the term didactic (“to teach”) with dissonance, referring to the tension or clash resulting in learners’ minds from the juxtaposition of two or more formal curricula or intentional teaching activities that differ in content.

Of note, didactic dissonance as described differs from the concept of the hidden curriculum (12). The hidden curriculum is a recognized set of ethical, moral and value-based influences that are informally passed to learners through observation in the clinical and classroom settings that have been shown to impact learner bias and future interactions (13–16). While the hidden curriculum in pain education might implicitly communicate to the learner that patients with chronic pain are exaggerating or fabricating their symptoms (17), didactic dissonance would be experienced by the learner when the approach observed in clinic differs from what was taught in the classroom. We are discussing didactic dissonance in the field of pain education. However, its occurrence in other fields, particularly those where external pressures are significant or emerging evidence has prompted a cultural shift in clinical care [e.g., antibiotics for a viral respiratory infection (18), hormone therapy in postmenopausal women (19)], highlights the potential usefulness of this approach both within and beyond the field of pain education.

Ideally, curricula are living and changing consistent with the evolution of knowledge and science. Realistically, both classroom and clinical teachings may become outdated or inconsistent with constantly changing medical literature. Therefore, didactic dissonance may be bidirectional such that modern best practices described in a formal classroom curriculum are contradicted in a clinical environment and vice versa. Additionally, learners may misidentify a discrepancy due to their misinterpretation of one or both of the curricula or practices. For expediency, the language in the remainder of this paper will primarily use the examples of a modern classroom curriculum and situations that diverge from that curriculum observed in clinical practice.

## Leveraging transformative learning theory

Transformative learning theory provides an intellectual framework for leveraging didactic dissonance as a tool for learning. Transformative learning theory, originally described by Mezirow in 1978 (20), is a theory of adult learning founded on the premise that adult learners adjust their worldview through critical reflection as they encounter new information.

**Figure 1:**
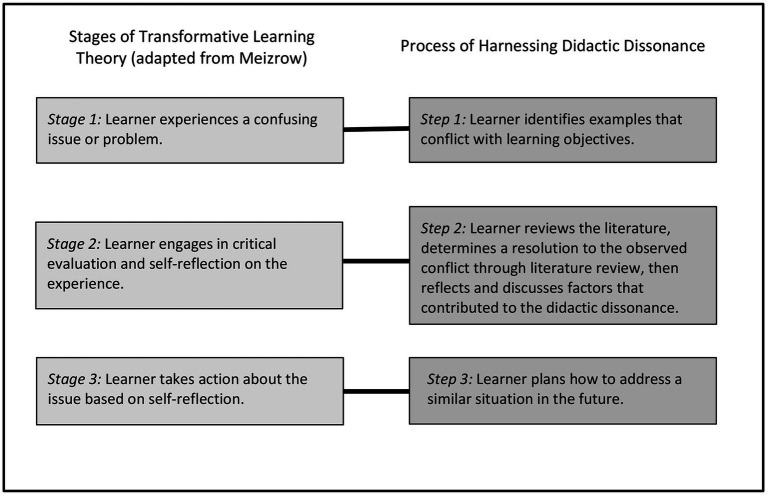
Stages of transformative learning mapped onto the proposed process of harnessing didactic dissonance. This figure shows the three main stages of transformative learning theory [from (20)] and how they correspond to the three steps of how to harness didactic dissonance.

Transformative learning can be thought of as occurring in three key stages (21): (1) encountering a disorienting and confusing problem or experience, (2) undergoing self-reflection and critical evaluation, and (3) establishing a new course of action, which involves planning, acquiring new skills, and incrementally testing and adopting new actions. These three stages of transformative learning can be mapped to a three-step process to use didactic dissonance to foster lifelong self-directed learning among medical practitioners (Figure 1).

## Harnessing didactic dissonance

We propose a three-step process to harness didactic dissonance as a learning tool, applying the key principles of transformative learning theory (Figure 1). The steps below have been intentionally designed with an eye toward ease of implementation and are based on preparing and facilitating a structured group discussion with learners.

Because didactic dissonance is based on identifying contradictions within two or more curricula, there could be a tendency for individuals or educators to think of these discrepancies as representing “faulty teaching.” This type of labeling rooted in intellectual arrogance, complacency, and closed-mindedness, is polarizing and can impair or arrest lifelong learning. Therefore, when harnessing didactic dissonance in medical education, particular care should be taken to promote a learning environment based on the intellectual virtues of intellectual humility, intellectual curiosity, and intellectual creativity, with a primary goal of fostering autonomous thinking (22). Throughout each of the three steps below, students should be encouraged to consider the motivations behind observed actions and clinical instruction, to have healthy skepticism that leads them to check their own beliefs, and to foster a spirit of curiosity that seeks out answers.

**Figure 2:**
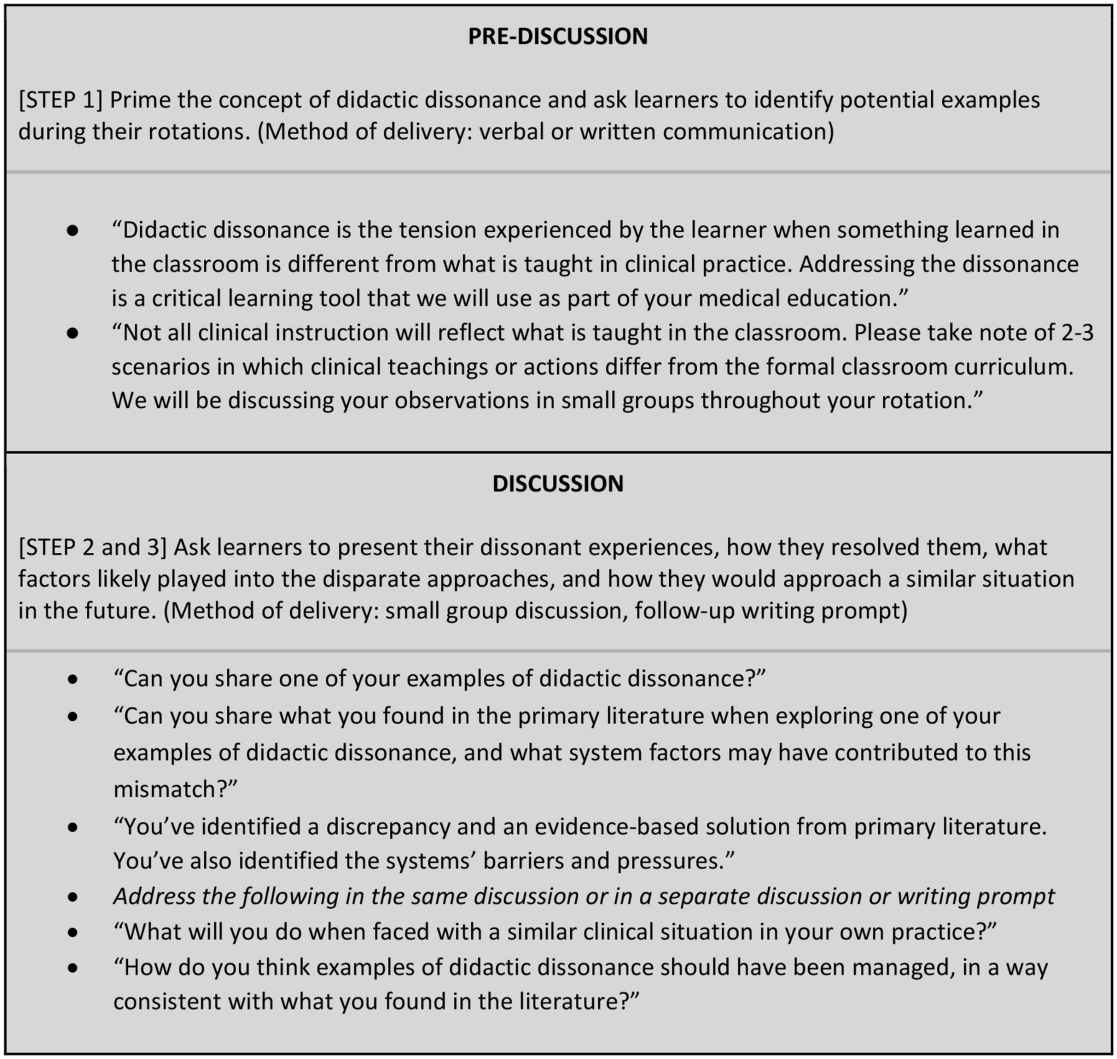
Didactic dissonance discussion guide. This discussion guide is a summary of sample language to help the educator address didactic dissonance as part of their curriculum. Of note, a subject matter expert in the fields of the particular rotation or in learning theory is not required.

Below, we describe our three-step process with a brief introductory description of the step and a suggested approach for implementation. Sample facilitator language in the form of a Discussion Guide is shown in Figure 2, and an example of a facilitated didactic dissonance group discussion is available in Appendix 1.

*Step 1: Prior to clinical rotations, introduce learners to the concept of didactic dissonance and prime them to identify examples in the clinical setting that conflict with classroom learning objectives.*

This step introduces the concept of didactic dissonance to learners and describes how it can be used as a tool for lifelong learning. Learners are encouraged to identify 2–3 examples of didactic dissonance where clinical teaching or observations differ from the classroom curriculum, and they are informed in advance that these examples will serve as the basis for future discussions. To promote the constructive learning environment we are seeking, medical educators can remind learners that the goal in identifying didactic dissonance is not to find fault, but rather to foster autonomous thinking, and that both classroom and teaching environments are subject to different factors that impact the real-world implementation of best practices.

This introductory or priming step can be implemented through an educator’s verbal presentation, in writing, or through email reminders before the start of rotations.

*Step 2: After clinical rotations, ask learners to pick one item on their list of examples and search the primary literature using principles of information mastery to determine a resolution to the dissonance. Learners should present their experience and path to resolution in a group setting, along with their reflection on the factors that created and perpetuated didactic dissonance.*

This step seeks to encourage learners’ intellectual curiosity as a way to resolve their experienced dissonance and help them better understand the underlying applied practice of medicine. It is important to be aware that judgment, accusations or nonverbal cues about one approach over another may create an unhelpful learning and professional environment and encourage intellectual arrogance, self-assured fault-finding, and closed-mindedness. Instead, the goal of the exercise is to create and model intellectual humility (e.g., “Maybe I should think about this differently”), intellectual curiosity (e.g., “What can I learn from this?”), and intellectual creativity (e.g., “I could try ‘X’ next time”).

It is essential to encourage learners to reflect on the factors that have created and perpetuated the observed examples of didactic dissonance. From the clinical perspective, examples could include short office visits (economic pressure), the desire to please patients (patient pressure), or outdated or misapplied knowledge. From the curricular perspective, factors could include individual biases and attitudes of curricular authors, adherence to national competencies, or the time burden required to create or update curricula.

This step can be implemented by scheduling a facilitated discussion post-rotation or at the end of the academic year in which students present, resolve, and reflect on the process.

*Step 3: Provide an opportunity for learner reflection and planning about how they will address the observed clinical scenario in future clinical or teaching experiences.*

This step creates an opportunity to translate theory into imagined and eventually actual practice. How will the learner, in future clinical and teaching contexts, implement the best practice from what they resolved from Step 2? How will the learner confront economic, patient, time, and other pressures?

By envisioning their future clinical and teaching practice, learners acknowledge the reality that no clinical practice or curriculum is perfect. Incorporating the intellectual virtues and becoming an autonomous thinker can help guide the learners toward a lifelong process of investigation, assessment, and reflection.

This step can be implemented by asking these reflective questions during the aforementioned discussion, in a separate follow up discussion, or through an individual writing prompt.

## Discussion

To our knowledge, this is the first description of a deliberate process to harness the divergent information that learners may encounter in the classroom and clinical settings as a force for learning. Our experience with didactic dissonance stems from pain education through the *Arizona Pain and Addiction Curriculum* (9), but we propose this process as one that can be applied to all domains of medical education, particularly those involving recent paradigm shifts or where challenges exist to implementing the best available science, such as addiction medicine, antibiotic stewardship, HIV pre-exposure prophylaxis, or vaccination.

We recognize that introducing a novel learning approach to an already crowded curriculum with competing priorities, overworked faculty and insufficient numbers of preceptors may face implementation challenges. These challenges may include concerns about feasibility or unintended consequences such as alienating clinical preceptors.

While the steps above were designed for implementation feasibility, a smaller, incremental approach may be a more achievable option for some programs. Schools could start small by linking the concept of didactic dissonance into already existing curricular elements, such as problem-based learning or humanities-in-medicine group discussions. A next step may be to carve out time for a structured small-group discussion, using Figure 1 as a Discussion Guide. The most comprehensive approach would be a longitudinal, multi-year incorporation of small group discussions and writing prompts to help learners internalize the process as part of their lifelong learning habits. Of note, it is not necessary for the discussion facilitator to have expertise in the specific clinical situation being explored; rather, the ideal facilitator would encourage critical thinking, skepticism, self-reflection, use of primary literature, and an environment of openness and curiosity.

Consideration of potential unintended consequences from any new process is key to success, and educators may be concerned that this practice could result in fault finding or finger pointing at an already short supply of preceptors. However, by acknowledging and exploring differences in curricular content, learners will likely develop a greater understanding of system factors that shape both the creation of classroom curricula as well as clinical practice. This awareness, combined with the information mastery to resolve observed differences and address similar situations in the future, should promote humility and a greater appreciation for the complexities of classroom teaching and clinical practice.

Parallel with the concern about finding adequate time to address didactic dissonance may be a cognitive bias to avoid direct identification of examples of divergent teaching content. However, didactic dissonance occurs whether time to address it has been allocated or not. Ignoring it would be a lost opportunity, and aiming to reduce or eliminate it would likely be more challenging and less feasible than the most favorable approach: adopting a deliberate educational process that leverages didactic dissonance to promote lifelong learning.

Particular care should be taken to promote a learning environment that fosters autonomous thinking and is based on the intellectual virtues mentioned previously in this manuscript: intellectual humility, intellectual curiosity, and intellectual creativity. Autonomous thinking is essential to becoming a lifelong learner, as it entails developing the cognitive skills and self-reflective inclination to critically assess one’s knowledge, attitudes, and practices (20).

The transformational learning process of harnessing didactic dissonance can be applied longitudinally, throughout residency, fellowship, and continuing education. And beyond the sphere of health education, this process provides a mechanism for effective lifelong learning. As learners we can go through life with sets of fixed knowledge that impair future learning, or we can bring a spirit of curiosity and openness, a willingness to change opinions, and a desire to go deeper and reconcile the differences we encounter so as to continually experience transformative learning.

## Data availability statement

The original contributions presented in the study are included in the article/Supplementary material; further inquiries can be directed to the corresponding author.

## Author contributions

AM and LV: project conception, drafting, and critical revision of manuscript. LK, HQ, and EH: project conception, critical review, and revision of manuscript. All authors contributed to the article and approved the submitted version.

## Funding

The funding for the Arizona Pain and Addiction Curriculum comes from Arizona’s State Opioid Response Grant. The funding for the publication fees is from the Arizona State Opioid Response Grant.

## Conflict of interest

The authors declare that the research was conducted in the absence of any commercial or financial relationships that could be construed as a potential conflict of interest.

## Publisher’s note

All claims expressed in this article are solely those of the authors and do not necessarily represent those of their affiliated organizations, or those of the publisher, the editors and the reviewers. Any product that may be evaluated in this article, or claim that may be made by its manufacturer, is not guaranteed or endorsed by the publisher.
