# Supplementary material for: Didactic dissonance—embracing the tension between classroom and clinical education
Source: Front Med (Lausanne). 2023 Jun 22;10:1197373. doi: 10.3389/fmed.2023.1197373 (PMC10323936; doi:10.3389/fmed.2023.1197373)
Supplement: Supplementary file 1 [file Data_Sheet_1.PDF]

APPENDIX 1: **Example of a Facilitated Didactic Dissonance Group Discussion**

Step 1: Prime the concept of didactic dissonance and ask learners to identify potential examples during their rotations. (Method of delivery: verbal or written communication)

[Educator]

"Didactic dissonance is the tension experienced by the learner when something learned in the classroom is different from what is taught in clinical practice. Addressing the dissonance is a critical learning tool that we will use as part of your medical education."

"Please note that not all clinical teachings and actions will reflect what is taught in the classroom. Please take note of 2-3 scenarios in which teachings or actions differ from the formal classroom curriculum. We will be discussing your observations in small groups throughout your rotation."

[Learner]

"List: 1) We learned in lecture that we should do all USPSTF screenings during a well check but the provider didn't and seemed rushed. 2) We learned in class to not rapidly taper off opioids but the provider did. Patient and provider quoted laws and regulations. 3) We were taught that benzodiazepines shouldn't be used due to increased risk of death; providers do prescribe benzodiazepines."

Step 2: Ask learners to pick one item on their list of examples and to search the primary literature and determine resolution to the dissonance. Learners should present their experience and path to resolution in a group setting, along with their reflection on the factors that created and perpetuated the didactic dissonance.

[Educator]

"Tell us about what you found in your literature review about how to manage patients on long-term opioid therapy. What do you think accounts for the different ways that practicing clinicians may manage patients on long-term opioid therapy?"

[Learner]

"I chose to research and reflect on Example 2. During my family medicine clerkship, I saw my preceptor tell a patient on oxycodone 60 mg/day that the patient can either be tapered off over the next four weeks or be referred to pain management due to a state law. I don't think an assessment of risks and benefits of long-term opioid therapy nor an assessment for opioid use disorder were performed. I remember that the Arizona Pain and Addiction Curriculum recommends you do both those things before making a decision about long-term opioid therapy."

"I confirmed what the Curriculum taught and that the evidence supports this. I know that it's difficult for a provider to manage because they have to balance evidence about the limited benefit of long-term opioid therapy with a desire to help the patient by providing what they are asking. I understand the frustration of the patient too. I wonder how comfortable the provider is with identifying opioid use disorder or whether he thinks that's the job of an addiction medicine specialist. And there wasn't much time, we were already 40 minutes behind."

Another group participant notes that additional time with the patient and additional provider training in pain and addiction would have likely led to a different clinical encounter. Multiple group participants discuss how to find the actual state law and misapplication of state laws and guidelines.

Step 3: Provide an opportunity for learner reflection and planning about how they will address the observed clinical scenario in future clinical or teaching experiences.

[Educator]
"When you encounter a similar situation in the future, how do you think you will address it? Are there any new skills that you think might help you address the situation in the way that you would like to when you encounter something similar in the future? Are there some small steps that you may be able to take to try out a different way of communicating with inherited patients who are receiving long-term opioid therapy?"

[Learner]
"In the future, I think I might apply what we learned in another lecture on motivational interviewing, because I wonder if that's a technique that might help in a similar patient interaction in the future. I'd probably make a template so I remember to ask about functional status, co-prescribed sedatives, substance use, and mental health and medical comorbidities to make sure I don't forget when I see a patient on long-term opioid therapy in the future, given time and other constraints of the clinic. And if I see a similar patient in the future, I'd offer a very gradual oxycodone taper option."

Group participants wonder how to stay up to date on state laws and regulations. Another group participant suggests signing up for listservs from the state regulatory board and public health department.
